# Management of Tubo-Ovarian Abscess in a Patient With Factor V Leiden Deficiency and High Body Mass Index (BMI)

**DOI:** 10.7759/cureus.67376

**Published:** 2024-08-21

**Authors:** Abbas Abuelhassan

**Affiliations:** 1 Obstetrics and Gynaecology, Ashford and St. Peter's Hospitals NHS Foundation Trust, Surrey, GBR

**Keywords:** conservative management, antibiotic therapy, penicillin allergy, factor v leiden deficiency, tubo-ovarian abscess

## Abstract

This case report details the clinical course, diagnostic challenges, and management of a 53-year-old female patient with a history of factor V Leiden deficiency, hypertension, and high body mass index (BMI), presenting with an acute tubo-ovarian abscess (TOA). The patient's medical history also included penicillin allergy, premenopausal bleeding, and two previous cesarean sections, adding complexity to her management.

Upon presentation, the patient exhibited symptoms of TOA, a severe complication of pelvic inflammatory disease (PID). Given her high BMI and surgical history, the risks associated with surgical intervention were significant. Consequently, a conservative approach with prolonged antibiotic therapy was chosen.

The diagnosis was supported by initial and follow-up CT scans, which revealed multiple fluid collections indicative of infection but did not suggest a safe access route for percutaneous drainage. The patient's penicillin allergy required a careful selection of antibiotics to ensure efficacy and avoid adverse reactions.

A multidisciplinary team comprising specialists from gynecology, microbiology, and radiology collaborated to devise and implement an effective treatment plan. This approach allowed for regular reassessment and adjustments to the therapeutic regimen. The patient received broad-spectrum antibiotics tailored to her specific needs, with the regimen prolonged due to the infection's severity and the high risk of surgical complications.

The patient's inflammatory markers, including C-reactive protein (CRP) levels, were closely monitored, guiding treatment adjustments. Over time, significant clinical improvement was observed, with a gradual decrease in CRP levels and symptom resolution.

This case underscores the importance of a tailored, patient-specific approach in managing complex TOA cases. It highlights the potential for conservative management with antibiotics in high-risk patients where surgical intervention poses significant risks. The successful outcome emphasizes the value of a multidisciplinary approach and individualized care in achieving favorable outcomes in TOA management.

## Introduction

A tubo-ovarian abscess (TOA) is a significant gynecological condition characterized by the formation of a pus-filled cavity involving the fallopian tube and ovary. Often a complication of pelvic inflammatory disease (PID), TOA represents a severe and potentially life-threatening infection that demands prompt medical intervention. The pathogenesis of TOA typically involves the ascent of polymicrobial organisms from the lower genital tract, leading to widespread inflammation and abscess formation [1]. The clinical presentation can be varied, ranging from subtle pelvic discomfort to acute abdomen with systemic signs of infection, posing diagnostic challenges [2].

The diagnosis of TOA can be challenging due to its variable presentation. Patients may exhibit a wide range of symptoms, from mild pelvic pain and discomfort to severe abdominal pain, fever, and signs of systemic infection such as tachycardia and hypotension. This variability necessitates a high index of suspicion and often relies on a combination of clinical, laboratory, and imaging findings for accurate diagnosis.

The standard management of TOA typically includes broad-spectrum antibiotics to cover the polymicrobial nature of the infection, which often involves anaerobes, aerobes, and gram-positive and gram-negative bacteria. In many cases, particularly those not responding to medical management or when there is a risk of rupture, surgical intervention such as abscess drainage or even more extensive surgery may be required. However, surgery carries risks, especially in patients with comorbid conditions or those who are otherwise poor candidates for operative management.

This case report aims to highlight the role of prolonged courses of antibiotics in the management of a patient with TOA who is at high risk for surgical interventions. By providing a detailed account of the patient's clinical presentation, diagnostic findings, treatment regimen, and outcomes, this report seeks to underline the importance of extended antibiotic therapy as a critical component in the management strategy for high-risk patients. Through this comprehensive case analysis, we aim to enhance understanding and promote effective clinical practices to improve patient outcomes [3,4].

Effective management of TOA, particularly in patients for whom surgery poses significant risks, requires a thorough understanding of the disease process, timely diagnosis, and a tailored approach to treatment. This report not only demonstrates the potential for successful medical management with prolonged antibiotic therapy but also emphasizes the importance of careful monitoring and follow-up to ensure complete resolution of the infection and prevent recurrence. By documenting this case, we aim to contribute to the body of knowledge guiding the management of TOA and improve clinical outcomes for this serious condition.

## Case presentation

The patient is a 53-year-old female with a significant medical history including premenopausal bleeding, two previous cesarean sections, hypertension managed with ramipril, factor V Leiden deficiency (heterozygous), penicillin allergy, and a BMI of 50.

Clinical presentation

On January 10, the patient reported a gradual onset of abdominal pain beginning at 20:00, initially localized to the right iliac fossa (RIF) and subsequently becoming generalized. The pain was described as crampy in nature with a severity of 10/10 and was associated with vomiting of recently ingested food. The patient felt feverish and had a bowel movement the previous day.

Physical examination

On examination, the patient was alert, awake, but in painful distress and appeared dehydrated with dry buccal mucosa. The abdomen was distended with marked tenderness. Vital signs revealed a temperature ranging from 37.7°C to 38.3°C and a heart rate of 126 beats per minute.

Initial management and investigations

Blood cultures were taken, and the patient was started on intravenous fluids and analgesia. A CT scan of the abdomen and pelvis on January 10 showed acute sigmoid diverticulitis with a small volume of free fluid in the pelvis. Intravenous antibiotics (gentamicin and teicoplanin) were initiated. Laboratory results revealed a C-reactive protein (CRP) blood test of 370 mg/L, a white blood cell (WBC) count of 11×10^9^/L, and a lactate of 2.4 mmol/L.

Progression and further management

**Figure 1:**
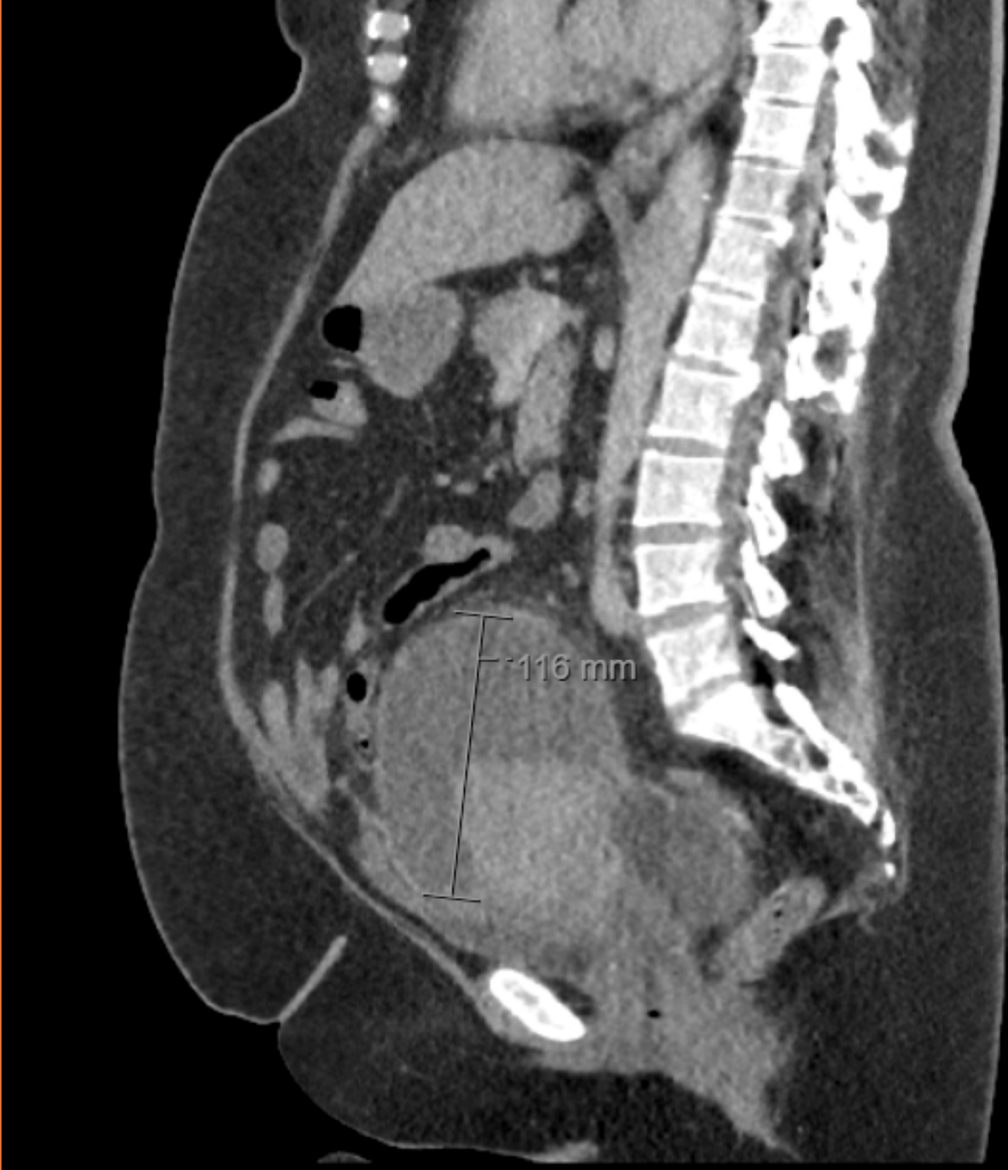
CT scan showing a 116 mm fluid collection (tubo-ovarian abscess)

By January 12, the patient's pain had improved but was still present, and abdominal tenderness persisted despite being afebrile. Antibiotics were continued, but CRP levels were rising, prompting the surgical team to order a repeat CT scan on January 15. The scan revealed several fluid collections within the abdominal cavity, the largest being an 11 cm collection surrounding the uterus, indicative of a TOA as shown in Figure 1. Due to the high risk associated with surgical drainage given her BMI and previous surgeries, a discussion with an interventional radiologist concluded that there was no safe access for drainage of the abscess; conservative management with antibiotics was chosen in consultation with a microbiologist.

On January 16, the patient remained afebrile but experienced intermittent tachycardia. CRP levels were decreasing. By January 17, she was clinically well with no further temperature spikes and a downtrending CRP and WBC as shown in Table 1. A follow-up CT scan was advised for 10 days from the initiation of intravenous antibiotics.

**Table 1:** The patient's CRP levels over time along with the normal reference range, providing a clear view of the inflammatory response and WBC counts during the course of treatment CRP: C-reactive protein; WBC: white blood cell

Date	CRP level (mg/L)	WBC (×10⁹/L)	Normal reference range
January 10	370	10.9	<10 mg/L, 4.5-11 (×10⁹/L)
January 11	650	13.5	<10 mg/L, 4.5-11 (×10⁹/L)
January 12	611	12.4	<10 mg/L, 4.5-11 (×10⁹/L)
January 13	522	13.4	<10 mg/L, 4.5-11 (×10⁹/L)
January 14	344	14.3	<10 mg/L, 4.5-11 (×10⁹/L)
January 15	354	16.1	<10 mg/L, 4.5-11 (×10⁹/L)
January 16	339	15.5	<10 mg/L, 4.5-11 (×10⁹/L)
January 17	276	13.9	<10 mg/L, 4.5-11 (×10⁹/L)
January 18	257	13.8	<10 mg/L, 4.5-11 (×10⁹/L)
January 20	187	12.5	<10 mg/L, 4.5-11 (×10⁹/L)
January 23	177	11.3	<10 mg/L, 4.5-11 (×10⁹/L)
January 25	232	13.4	<10 mg/L, 4.5-11 (×10⁹/L)
January 26	236	9.3	<10 mg/L, 4.5-11 (×10⁹/L)
January 27	210	9.1	<10 mg/L, 4.5-11 (×10⁹/L)
February 2	79	6.9	<10 mg/L, 4.5-11 (×10⁹/L)
February 12	5.6	N/A	<10 mg/L, 4.5-11 (×10⁹/L)
February 20	4.9	N/A	<10 mg/L, 4.5-11 (×10⁹/L)

Extended antibiotic therapy

On January 18, the patient showed clinical improvement with no fever spikes. However, a discussion with the microbiology team suggested that without surgical drainage, the abscess was unlikely to resolve and required a prolonged course of antibiotics. Consequently, the patient was started on teicoplanin 800 mg OD IV, metronidazole 400 mg TDS, and ciprofloxacin 750 mg BD for six weeks. She was planned to be discharged home with a peripherally inserted central catheter (PICC) line for continued IV antibiotics and scheduled for a repeat CT scan in 10 days.

The patient remained hospitalized until January 21 due to declining renal function, suspected to be secondary to teicoplanin or sepsis. A microbiologist recommended switching to levofloxacin and metronidazole and repeating the CT scan. The scan on January 23 showed a stable multilobular thick-walled fluid collection measuring 10.2×10.5 cm and a slightly increased collection in the pouch of Douglas.

**Figure 2:**
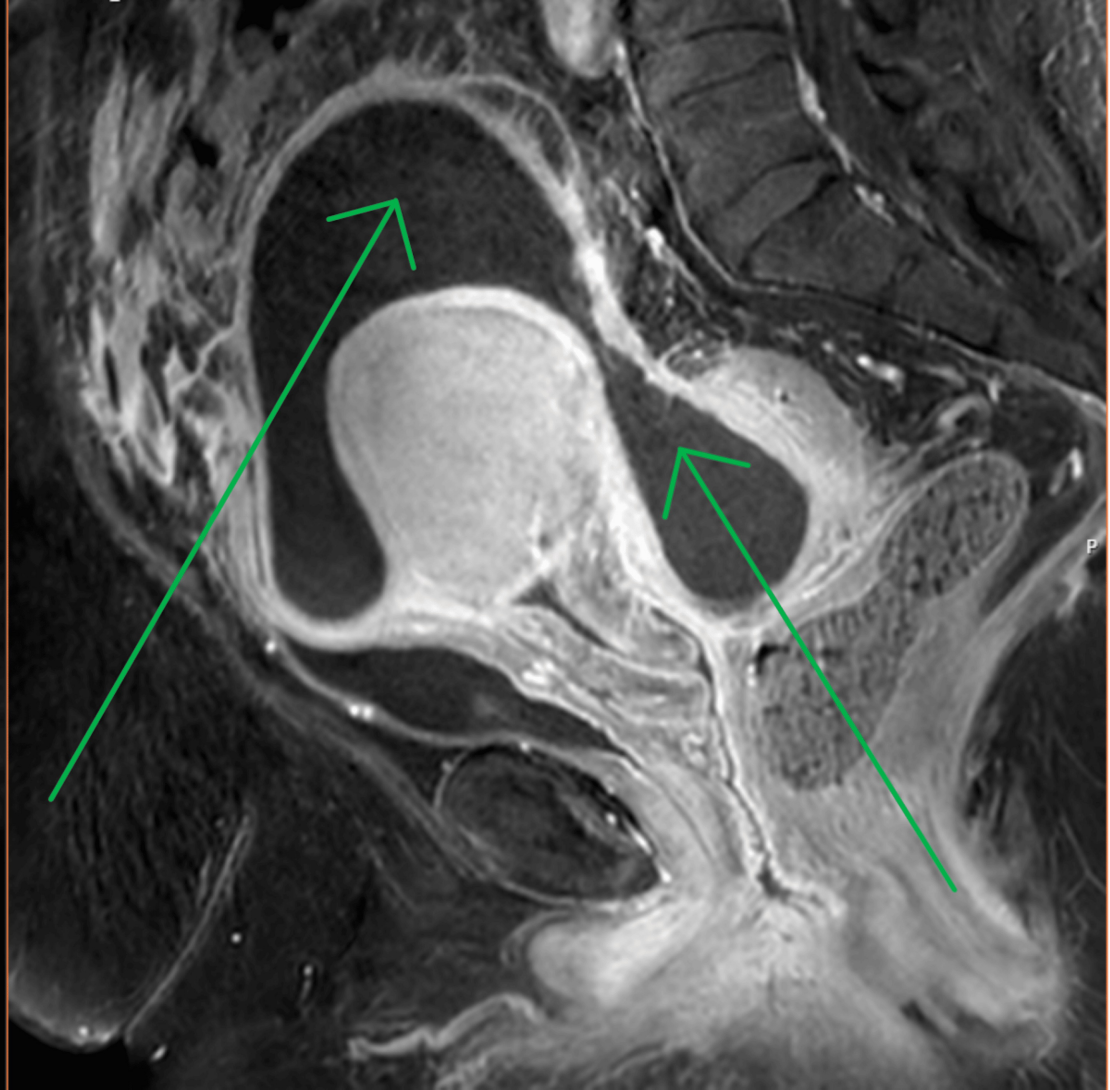
MRI demonstrating a superior and anterior collection to the uterine fundus and a smaller collection in the rectouterine space

By January 28, renal function had improved, and the patient was clinically well. An MRI on January 30 revealed a superior and anterior collection to the uterine fundus and a smaller collection in the rectouterine space, communicating and measuring 11.8×8.4 cm, along with a right adnexal pyosalpinx about 5.6×3.3×5.3 cm as shown with greens arrows in Figure 2.

Discharge and follow-up

The patient was discharged home with oral levofloxacin 500 mg BD and metronidazole 400 mg TDS. Her case was discussed in the multidisciplinary team meeting (MDT) on February 5, and a transvaginal ultrasound scan (TVUS) was advised in eight weeks. However, during the acute gynecology clinic visit on February 12, microbiologists recommended continuing antibiotics for an additional two weeks and performing a TVUS in two weeks.

Outcome

**Figure 3:**
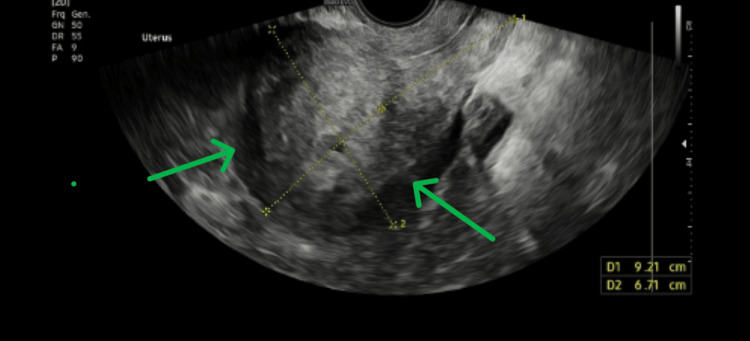
TVUS showing no obvious pelvic collections TVUS: transvaginal ultrasound scan

A TVUS on March 7 showed no obvious pelvic collections, cysts, or masses, indicating the resolution of the abscess (Figure 3).

## Discussion

TOA is a severe complication of PID that requires timely and effective management to prevent significant morbidity and mortality. Standard treatment typically involves a combination of antibiotics and, when necessary, surgical drainage. However, this case report discusses a unique scenario where conservative management with a prolonged course of antibiotics was chosen due to the patient's high BMI and history of previous cesarean sections, which posed significant risks for surgical intervention.

The initial diagnosis was complicated by the patient's complex medical history, including factor V Leiden deficiency, hypertension, and penicillin allergy. These factors necessitated a careful selection of antibiotics and close monitoring of the patient's response to treatment. Recent studies have shown that conservative management of TOA can be successful in selected patients, particularly when surgical risks are high and appropriate antibiotics are used effectively [5].

The decision to manage this patient conservatively was supported by the findings of initial and follow-up CT scans, which revealed multiple fluid collections indicative of infection but did not suggest a safe access route for percutaneous drainage [6].

Throughout the patient's course of treatment, a multidisciplinary approach was essential. Collaboration between gynecology, microbiology, and radiology teams facilitated regular reassessments and adjustments to the treatment plan, ultimately leading to the resolution of the abscess without the need for surgical intervention. This approach aligns with evidence from recent studies, which emphasize the importance of individualized patient care and the potential for conservative management in high-risk patients [7].

This case underscores the importance of individualized patient care. The prolonged antibiotic regimen, although challenging in terms of compliance and monitoring, was tailored to the patient's specific needs and medical history. Regular follow-up and imaging confirmed the gradual resolution of the abscess, highlighting the potential for conservative management in similar high-risk patients. While surgery remains a common treatment for TOA, there is growing evidence supporting the efficacy of antibiotic therapy alone in carefully selected cases [5].

This case demonstrates the feasibility of managing a complex TOA conservatively with a prolonged course of antibiotics, particularly when surgical intervention poses significant risks. The successful outcome emphasizes the value of a multidisciplinary approach and the importance of individualized care in the management of TOA.

## Conclusions

This case illustrates the complexities and challenges involved in the conservative management of a TOA, particularly in a patient with significant comorbidities such as high BMI, previous cesarean sections, hypertension, factor V Leiden deficiency, and a penicillin allergy. Despite the initial lack of safe access for surgical drainage as confirmed by interventional radiology, a multidisciplinary approach involving gynecology, microbiology, and radiology teams enabled the successful resolution of the abscess through a prolonged course of tailored antibiotic therapy.

Key aspects of this case include the careful monitoring of CRP levels and renal function, adjustments in antibiotic regimens, and the importance of regular imaging to track the progress of the abscess. The patient's outcome highlights the potential for non-surgical management in similar complex clinical scenarios, emphasizing the value of a collaborative, patient-centered approach to care.

This case contributes valuable insights into the feasibility and effectiveness of conservative management strategies for TOA, providing a reference for future cases where surgical intervention may pose significant risks.
